# Fungal Bezoar: A Rare Cause of Ureteral Obstruction

**DOI:** 10.1155/2017/6454619

**Published:** 2017-07-19

**Authors:** Nabil Zeineddine, Wissam Mansour, Sandy El Bitar, Marco Campitelli, Neville Mobarakai

**Affiliations:** ^1^Hofstra Northwell School of Medicine at Staten Island University Hospital, 475 Seaview Avenue, Staten Island, NY 10305, USA; ^2^Northwell Health at Staten Island University Hospital, 475 Seaview Avenue, Staten Island, NY 10305, USA

## Abstract

A 52-year-old male, with diabetes mellitus and alcoholic liver disease, presented to the Emergency Room for right flank pain of 3 days' duration, associated with dysuria. Physical examination revealed right flank tenderness with fever and hypotension; laboratory findings showed acute kidney injury and large blood and leucocytes in the urine. A CT abdomen and pelvis showed hydronephrosis of the right collecting system of a horseshoe kidney with air and hyperdense debris in the renal pelvis. Patient was treated for multisensitive* Proteus mirabilis* emphysematous pyelonephritis, and a right nephrostomy tube was inserted. Symptoms recurred in 4 weeks, and repeated urine culture grew* Candida albicans* and CT scan showed same high density material within the right moiety of the horseshoe kidney. Patient underwent ureteroscopy, and a white fluffy material was aspirated from the right renal pelvis. Pathology of the aspirate confirmed the presence of fungal balls. Patient was given 2 weeks of oral fluconazole. Fungal pyelonephritis is unusual and difficult to treat.* Candida* species is responsible for the clear majority of the cases. A fungus ball should be managed with surgical and medical therapy. This patient had an endoscopic procedure to remove the fungus ball and received fluconazole. His symptoms resolved and urine culture was done before termination of the treatment was negative.

## 1. Case Presentation

**Figure 1:**
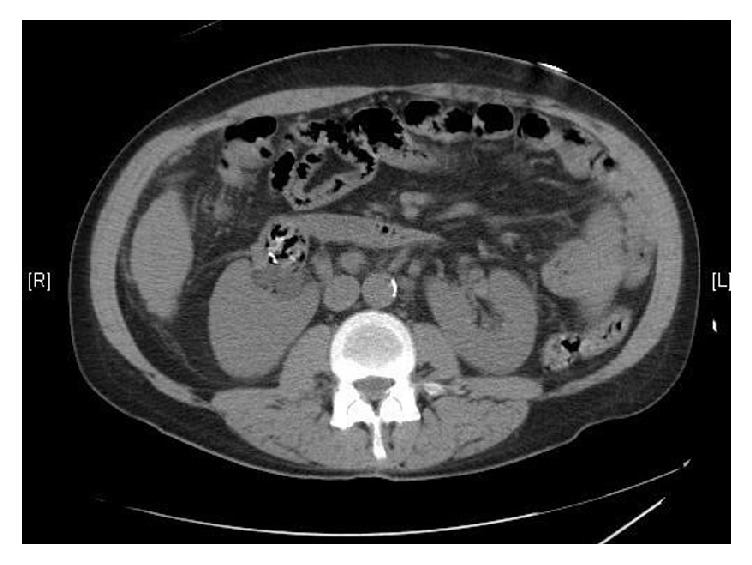
Abdominal CT scan, transverse view. Right renal pelvis collection (with large amount of air and hyperattenuating debris).

A 52-year-old male with past medical history of diabetes mellitus, horseshoe kidney, and alcoholic liver disease presented for right sided flank pain of 3 days' duration. Pain was radiating to the groin area, associated with dysuria and dark urine. The patient also noted episodes of chills at home with no fever. Home medications included long acting and sliding scale insulin coverage, spironolactone, and thiamine. Upon presentation patient's vital signs showed a blood pressure of 87/51 mmHg and a heart rate of 89 bpm. Intravenous fluid resuscitation was started with lactated ringer and ceftriaxone was administered for presumed pyelonephritis. Initial blood work revealed leukocytosis of 18,320/*μ*l with 90% neutrophils, lactic acid level of 3 mmol/L, and an increase in serum creatinine to 4.82 mg/dL from a normal baseline. Noncontrast CT of the abdomen and pelvis was significant for a horseshoe kidney with mild to moderate hydronephrosis of the right moiety collecting system containing large amount of air and hyperattenuating debris in the renal pelvis (Figure 1). Findings were suggestive of emphysematous pyelitis with an element of obstruction, related to the high density material within the right renal pelvis. Following fluid resuscitation with 3 L of lactated ringer the patient remained hypotensive with a mean arterial pressure of 55 mmHg requiring norepinephrine for pressure support. Patient was admitted to the intensive care unit (ICU) for septic shock secondary to obstructive emphysematous pyelitis. He then underwent an urgent right sided percutaneous nephrostogram demonstrating a large filling defect in the right renal collecting system. A nephrostomy pigtail drainage catheter was placed to relieve the obstruction. In the ICU, the patient's antibiotics were switched to Meropenem for broader empiric antibiotic coverage. On day 2 patient's serum creatinine improved to baseline and white count decreased to 7,100/*μ*l. Urine culture grew multisensitive* Proteus mirabilis*. Antibiotic coverage was deescalated to ciprofloxacin; norepinephrine was discontinued and patient was transferred to floor. On day 3 patient was discharged on a 4-week course of ciprofloxacin and instructions to follow up with urology for evaluation of the renal pelvis obstruction. Following discharge patient failed to follow up with urology clinic and returned to the hospital on week 5 with worsening flank pain and purulent discharge from the nephrostomy bag. Vitals on presentation were stable. CT of the abdomen and pelvis with IV contrast revealed right side perinephric fat stranding with mild hydronephrosis. It again noted a bezoar-like collection of hyperattenuating material and gas pockets in the renal pelvis. Patient was started on broad spectrum antibiotics for suspected relapsing pyelonephritis. The nephrostomy catheter was replaced after a nephrostogram suggested malpositioning. Urine culture from the purulent drainage grew* Candida albicans*. Blood culture was repeatedly negative. Based on the culture results, the obstructing ureteral bezoar was suspected to be a fungus ball and a daily dose of 400 mg of oral fluconazole was started. The patient then underwent an antegrade ureteroscopy revealing an organized collection. Following several breaking maneuvers areas of fluffy whitish tissue were noted and successfully suctioned. A nephroureteral stent was placed with closure of the nephrostomy. Pathology analysis confirmed the presence of multiple fungus balls admixed with acute and chronic inflammatory cells. The patient tolerated the procedure well and was discharged home following a 5-day hospital stay on oral fluconazole 400 mg daily for a total of 14 days. A negative urine culture and a CT scan were done before termination of treatment confirmed resolution of the infection.

## 2. Discussion

Fungal infections of the upper urinary tract are relatively uncommon. The increased use of antibiotics, corticosteroids, and indwelling urinary catheters have been implicated as risk factors for these infections predominantly caused by* Candida *spp. [1].

The presence of candiduria may reflect diverse disease states, including lower urinary tract colonization, superficial lower urinary tract infection, invasive pyelonephritis, and, rarely, fungal ball obstructing the ureters [2]. Fungal ball, also known as fungal bezoar, is a colonization of a cavity by an aggregate of fungal mycelia. This case describes* Candida albicans* fungus ball in the ureter, a rare clinical entity that usually requires high index of suspicion and early recognition in order to overcome the challenging treatment that necessitates both medical and surgical approaches [3].

In the renal parenchyma, fungal ball causes pyelonephritis and cortical abscess, while, in the draining system, the ball can lead to obstructive uropathy, most commonly at the level of ureteropelvic junction. Most reported cases of fungal ball are secondary to disseminated candidiasis, but literature also reveals that this type of disease, as in our patient, can be representative of an ascending process [4]. A workup including blood culture and if positive ophthalmological exam and echocardiogram to rule out systemic candidiasis is required in the presence of* Candida* sp. fungus ball [5]. The lack of evidence of disseminated fungal infection in our patient supports the hypothesis of ascending infection, despite the absence of previous urinary catheterization.

Candiduria frequently coexists with or follows bacteriuria [6]. In our patient, the presence of the fungus ball at the time of diagnosis of* Proteus mirabilis* pyelonephritis refutes the idea that candiduria followed the bacteriuria. Probably, the presence of the fungal ball leads to obstructive uropathy facilitating bacterial infection, resulting in* Proteus mirabilis* pyelonephritis.

Previous reports on fungal urosepsis have identified diabetes mellitus and immunosuppression as major risk factors for this disease [7, 8].

A conclusive diagnosis of fungal bezoar requires clinical, laboratory, and imaging studies. Clinically, abdominal pain and hematuria along with signs and symptoms of pyelonephritis and acute kidney injury constitute the most commonly encountered presentations [9]. Growing a microbiological culture of the pathogen from the urine and determining predisposing factors facilitate diagnosis. However, radiological confirmation of fungus balls is not conclusive and may be imitated by other pathological structures: blood clots, urinary calculi, air bubbles, inflammatory lumps, and epithelial tumors [10]. In our patient, the accessibility of the bezoar by ureteroscopy facilitated diagnosis by providing histopathological proof of the disease.

Regarding treatment, drainage by nephrostomy or ureteral stent is usually required. The most described therapeutic approach is urinary drainage by a nephrostomy and/or a ureteral catheter with systemic and local antifungal administration [11]. Nephrostomy also provides access to percutaneously remove the fungus ball and manually drain it via ureteroscopy [9]. In our patient, endoscopic removal of the fungus ball was successful, coupled with fluconazole treatment initiated prior to the procedure and given only systemically for 2 weeks' duration. Fluconazole in this case, besides being active against* Candida albicans*, achieves high level in urine. All other azoles as well as amphotericin B lack this feature [12]. Imaging to prove resolution of the obstruction is necessary prior to termination of antifungal therapy.

Prognosis of fungal urosepsis, particularly with the presence of fungal ball, is usually guarded owing to its association with disseminated disease. In our patient, absence of disseminated disease and prompt initiation of therapy resulted in good clinical outcome with cure of the fungal bezoar.

## 3. Conclusion

Fungal ball in the upper urinary tract is a rare entity. Early recognition is essential to start aggressive therapy including bypass of the obstruction, extraction of the mass, and initiation of antifungal therapy. Delay in diagnosis can lead to disseminated disease which usually carries worse prognosis. This case describes a* Candida albicans* ureteral bezoar that was successfully treated endoscopically along with 2 weeks of oral fluconazole given at a dose of 400 mg per day. Clinicians should be aware that antifungals are not sufficient for the resolution of the infection and that dissemination of the infection should be ruled out by appropriate laboratory and imaging studies.
